# The effect of classroom size and ceiling height on college students’ learning performance using virtual reality technology

**DOI:** 10.1038/s41598-024-65754-2

**Published:** 2024-07-03

**Authors:** Yalin Zhang, Chao Liu, Jiaxin Li, Xiaotong Jing, Jing Shi, Weijun Gao

**Affiliations:** 1 Innovation Institute for Sustainable Maritime Architecture Research and Technology, Qingdao University of Technology, Qingdao, 266033 China; 2 Faculty of Environmental Engineering, The University of Kitakyushu, Kitakyushu, 808-0135 Japan

**Keywords:** VR, Room size, Ceiling hight, EEG, HRV, Learning performance, Environmental social sciences, Engineering

## Abstract

The physical characteristics of classrooms can significantly impact the physical and mental health as well as the learning performance of college students. This study investigates the effects of classroom size and ceiling height on learning performance using virtual reality technology. Four classroom settings were created: two small classrooms (40.5 m^2^) with ceiling heights of 3.0 m and 3.9 m, and two large classrooms (62.1 m^2^) with ceiling heights of 3.9 m and 4.8 m. 34 students participated in task tests while their subjective evaluations and physiological data were recorded. Results showed higher subjective ratings in larger classrooms with the same ceiling height. Classroom size did not significantly affect task test scores. However, there is a significant difference in Task test scores for ceilings of different heights with the same size classroom. The task test improved by 17.3% in the Big and High Room (BHR) and by 20.1% in the Small and Low Room (SLR). Physiological data revealed significant effects of ceiling height, with HRV-nLF/nHF and EEG-β power increasing by 26.5% and 53.9% in BHR, and by 10.7% and 22.8% in SLR, respectively. This study concludes that classroom size and ceiling height plays a crucial role in learning performance and provides insights for classroom design. It also establishes a framework for future research on the interplay between heart rate variability, EEG, and learning performance.

## Introduction

Many experiments have been devoted to exploring the effects of thermal as well as light environments on human beings^1–3^. And now, the rising focus on ergonomics has led researchers to explore numerous factors that may affect human comfort and learning performance. The room size and ceiling height affect visual comfort^4–6^. Many studies have explored the relationship between classroom size and ceiling height with comfort and efficiency^7–9^.

Sundstrom E conducted stress tests on participants in both large and small rooms. The results indicated that crowding in small rooms resulted in a significant 25.3% increase in stress levels^10^. Large rooms with ample natural light have been found to elicit improved psychological and physiological responses^11^. Similar findings were found in a study by Nagayasu and Nakamura et al.^12^ where a questionnaire showed that participants felt comfortable in large rooms and uncomfortable in small rooms. Additionally, a study^13^ found that people tend to experience more positive emotions in spacious environments compared to closed and narrow ones. Cruz-Garza J G et al. conducted a study in which participants completed cognitive tests in virtual classrooms of varying sizes. The results indicated that participants exhibited superior cognitive performance in larger rooms, as evidenced by significant differences in EEG data^14^. In their study, Beckers et al.^15^ found that participants showed a preference for large rooms when engaging in collaborative learning activities, while they preferred small rooms for individual learning. Pejtersen et al.^16^ found that participants expressed greater satisfaction with office rooms that were larger in size and had higher ceilings. Fischl and Garling^17^ found that mental health is influenced by ceiling height. The results of the study showed that when people were exposed to high ceilings, they produced more positive emotional responses such as ‘happy’, ‘comfortable’ and ‘interesting’^18^. It has been suggested that ceiling height comfort is related to the posture and height of the individual; with a ceiling of 1.5 m, pressure from the ceiling can be uncomfortable when volunteers are sitting in a chair, but when reclining, this height is considered comfortable^19^. Haner^20^ found that open office spaces with higher ceiling heights can enhance innovative thinking and productivity. In a study conducted by Read M, four rooms were arranged with varying ceiling heights. The findings indicated that participants exhibited improved collaborative behaviors in larger classrooms with higher ceilings^21^. According to Mahat et al.^22,23^ individuals in classrooms with lower ceilings demonstrated improved learning performance, while those in classrooms with higher ceilings exhibited enhanced creativity, sense of participation, and cooperation. Moore, G.T. et al.^24^ concluded that ceiling heights below 8 feet may lead to more restrained and focused behavior, whereas ceiling heights above 8 feet may lead to more socially active behavior.

The above findings suggest that having the right room size and height can lead to both physical and psychological satisfaction, as well as enhance learning performance^25–28^. However, the current related research also has some limitations. The experiments mentioned above primarily relied on subjective data collected through questionnaires^15–17^. However, there is a need for objective data, specifically physiological indicators, to further validate learning performance. These days, an increasing number of research have utilised physiological data to study psychological changes in participants^29–33^. Cole H. et al.’s study^34^, which monitored the EEG signals of forty males, eventually demonstrated that variations in the participants’ mood and attentional levels were reflected by corresponding changes in their EEG signals. As a result, Ray W and Laufs H et al.^35,36^ conducted experiments for investigating into the trends connected to these changes in EEG data. It was found that the beta frequency of the EEG indicated cognitive activity in the brain, while the alpha frequency indicated a neurological baseline of “lack of attention”. In addition, heart rate variability has also been used in experiments to study how the environment affects the intensity of attention, and Carmen L^37^, in order to investigate and analyse the effects of warm and cool classroom spaces on the cognitive functions of attention and memory functions of college students, showed that there was a significant difference in the performance of college students in different classroom spaces, and that there was a significant correlation between the HRV LF/HF and the intensity of attention. Additionally, Yao^38^ and Liu et al.^39^ found that there were significant differences in HRV between the environments, with participants’ LF/HF = 1 *(p* < 0.01) in comfortable hot environments and > 1 (*p* < 0.01) in colder and warmer environments. Based on these findings, the authors proposed that LF/HF values could be used as a neurophysiological evaluation criterion in the future to measure thermal comfort in humans. Furthermore, many studies examining the impact of scale on performance have encountered limitations due to the requirement of constructing the venue on-site or finding an existing physical space. This constraint arises from the challenges associated with managing real space. Virtual Reality (VR) technology allows for the rapid creation of immersive visual experiences by simulating a wide range of spaces and their environmental elements. Previous studies have shown that there is no notable distinction in the psychological and physiological test results of participants in virtual reality and physical environment^40^. As a result, virtual reality technology has become increasingly popular in various research disciplines for conducting experiments^41–44^. In particular, VR technology is also frequently used to explore learning performance^45,46^, with Schiller^47^ setting up immersive virtual scenarios via VR as a way of exploring the impact of lecturer’s voice quality on student learning performance. There is also a section of research that combines VR with classroom learning, further validating the feasibility of VR technology in exploring student learning performance^48,49^.

This study employed VR technology to investigate the impact of classroom sizes and ceiling heights on learning performance. Virtual classrooms with varying dimensions are created, and participants wear VR glasses to complete task tests for subjective evaluation and objective physiological data collection. This study aims to enhance learning performance and promote physical and mental well-being among college students. It provides theoretical support for the development of an optimal learning environment.

## Methodologies

This study utilized VR technology to generate classroom spaces of different sizes and ceiling heights. Participants were tested on learning tasks in the virtual space. Data on psychological (questionnaires) and physiological indicators (HRV and EEG) were also recorded so as to explore the pattern of the effects of different classroom sizes and ceiling heights on participants’ learning performance. The experiment complied with the Declaration of Helsinki and was approved by the Ethics Committee of Qingdao University of Technology.

### Experimental scene design

Unity (version: 2021.3.0f1, website link: https://unity.com/cn) software were used to perform computer modeling based on a real typical Chinese classroom. The virtual classroom was 9000 mm × 7000 mm × 3800 mm. Participants wore VR glasses, the above model was projected in the VR glasses, and participants were seated at designated locations in the virtual classroom. The test questions appeared on the blackboard of the classroom. During the experiment the participants sat at a table and followed the prompts to complete the experiment while the EEG as well as ECG (electrocardiogram) data of the participants were collected.

**Figure 1:**
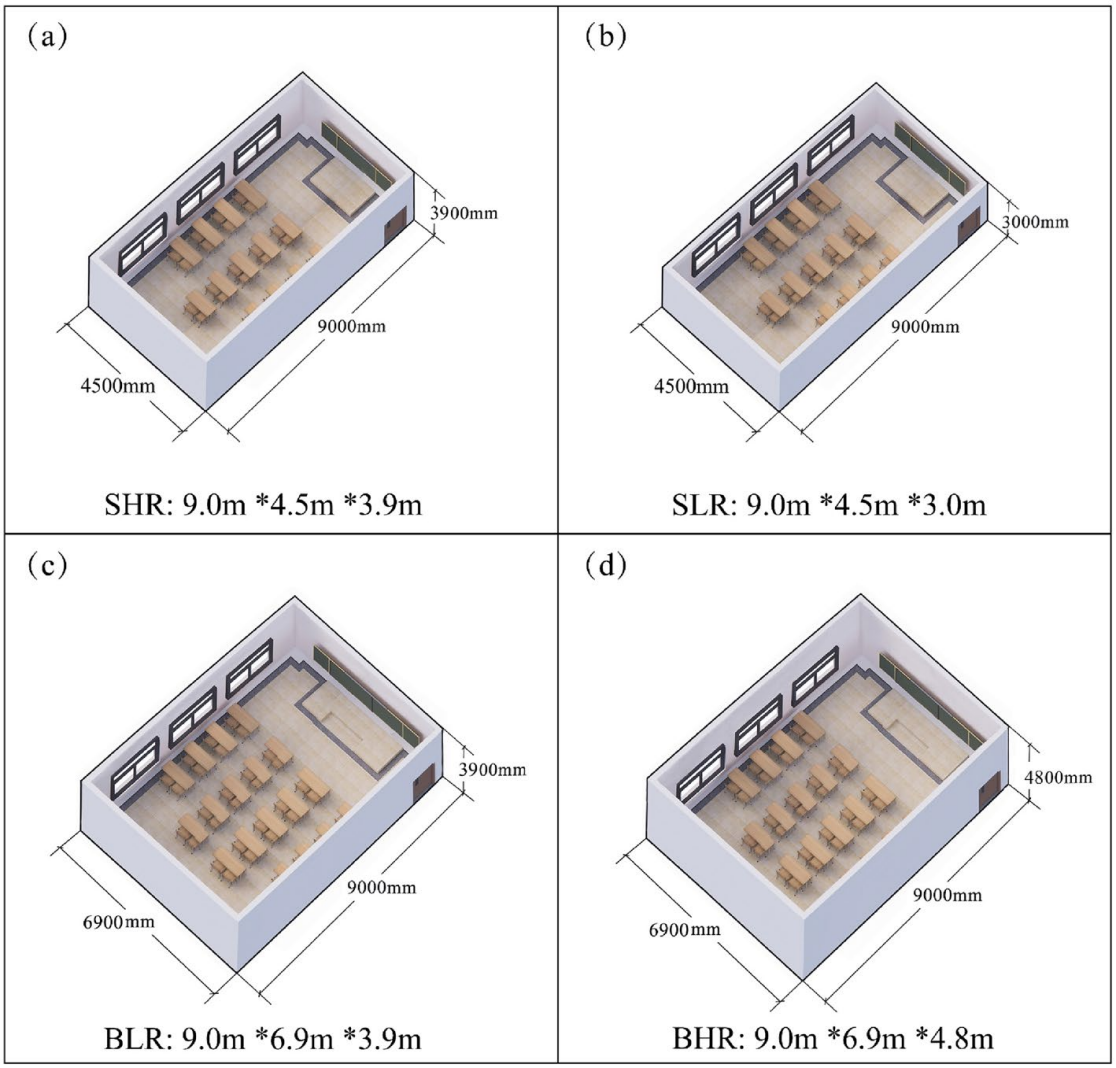
(**a**–**d**) VR models of the four scenes.

Figure 1a–d shows the VR models of the four experimental scenarios. The length of all four classroom models was kept constant at 9.0 m. Classroom sizes and ceiling heights were varied by adjusting the width and height of the models, respectively: (a) Small and High Room (SHR): 9.0 m*4.5 m*3.9 m; (b) Small and Low Room (SLR): 9.0 m*4.5 m*3.0 m; (c) Big and Low Room (BLR): 9.0 m*6.9 m*3.9 m; (d) Big and High Room (BHR): 9.0 m*6.9 m*4.8 m. All of the models were furnished identically, differing only in room size and ceiling height.

### Experiment equipment

The study was conducted using VR technology and physiological indicator acquisition technology. Air conditioning was used to control the room temperature and humidity environment consistently during the experiment, and a temperature and humidity meter was used to monitor the parameters. The equipment used in the experiment is shown in Table 1.

1. Head-mounted display: VR technology enables the efficient creation of extensive virtual environments, incorporating realistic environmental elements to deliver an immersive visual experience. Participants utilised a Head Mounted Display (HMD) to engage in simulated and real-life scenarios. The HMD device used in the study was an HTC Vive. It had a resolution of 2160 1200 pixels, a refresh rate of 90 Hz, and a field of view of 110. The device was calibrated before the experiments to ensure consistency in colours, light sources, and viewing angles.
2. Physiological data collection equipment: The human body’s daily functioning involves processing external information through the central nervous system and generating associated behavioural processes. These processes can be influenced by external environmental factors. Hence, it can be assessed through the measurement of pertinent physiological parameters. EEG signals offer prompt feedback on environmental changes in the body’s surroundings, while ECG signals serve as a significant indicator of autonomic nervous system activity during learning. This study utilised EEG-related indicators and heart rate variability as physiological measures to assess the participants’ learning activities. The participants in the study utilised an EEG device (Emotiv EPOC Flex Gel Sensor Kit) and an ECG device (Healink-R211B) to record their brain and heart activity. The raw data from the EEG and ECG recordings were collected for analysis.
3. Environmental detection equipment: The study was conducted from September to October 2022, with daytime temperatures ranging from 27 to 28 °C. According to a study conducted by Stone et al.^50^, participants expressed greater comfort when situated indoors within a temperature range of 24–26 °C, The indoor temperature was maintained at 25.5 °C using an air conditioner. The temperature and humidity detector recorded an average temperature of 24 ± 0.5 °C and an average humidity of 50% ± 5%. The typical thermal insulation value for indoor clothing worn by individuals ranges from 0.4 to 0.5 clo.

**Table 1:** Experiment equipment.

Useage	Type	Test program	Parameter	
			Resolution	View
Head mounted display	HTC Vive	Immersive VR scene build	1080 × 1200 per eye	110°
			Sampling rate	Bandwidth
Physiological signal monitoring	Emotiv EPOC Flex Gel Sensor Kit	EEG	128 Hz	0.16–43 Hz
	Healink-R211B	ECG	400 Hz	0.6–40 Hz
			Precision	Rage
Environment monitoring	iBEM 3G19	Temperature	± 0.5℃	− 20 ~ 70℃
	iBEM 3G19	Relative humidity	± 3%	0 ~ 100%

### Experiment participants

This study conducted a review of similar experiments to determine the appropriate sample size for the experiment. Li and Wu^51^ conducted a VR simulation experiment with 30 students, averaging 23.5 years old. Abd-Alhamid^52^ recruited 32 participants, including 19 females, for a similar experiment. This study aimed to include a diverse range of university students while minimizing the impact of gender and body mass index (BMI) on the experiment’s outcomes. A total of 34 participants (18 female and 16 male) were selected from Qingdao University of Technology, with an average age of 21.6 years. Among the participants, 76% had a normal BMI, as indicated in Table 2.

**Table 2:** Statistical table of the characteristics of the participants.

Characteristics	Number	Ratio (%)	
Gender	Male	16	47
	Female	18	53
Age	18–21	19	56
	22–25	15	44
BMI	Thin (BMI < 18.5)	3	9
	Normal (18.5 < BMI < 23.9)	26	76
	Fat (BMI > 24)	5	15

All participants had to meet health criteria upon enrollment, including the absence of sensory fever, color blindness, and other major illnesses. To mitigate the impact of variations in visual acuity, participants needed to have unaided or corrected visual acuity of 1.0 or higher. Furthermore, less than 15% of participants reported prior experience with VR within the past year prior to the commencement of the study. All candidates received a briefing on the experimental setup, procedures, questionnaire requirements, and task test, similar to the formal experiment, during the pre-experiment. In the pre-experiment phase, individuals experiencing symptoms such as dizziness, nausea, or other conditions that were incompatible with the virtual reality environment were excluded from the final subject pool. Participants were instructed to maintain a regular diet and sleep pattern one week prior to the experiment to ensure their physical and mental well-being. Additionally, they received training for the task test to prevent any potential inaccuracies caused by unfamiliarity with the task. Informed consent was obtained from all participants.

### Experiment process

**Figure 2:**
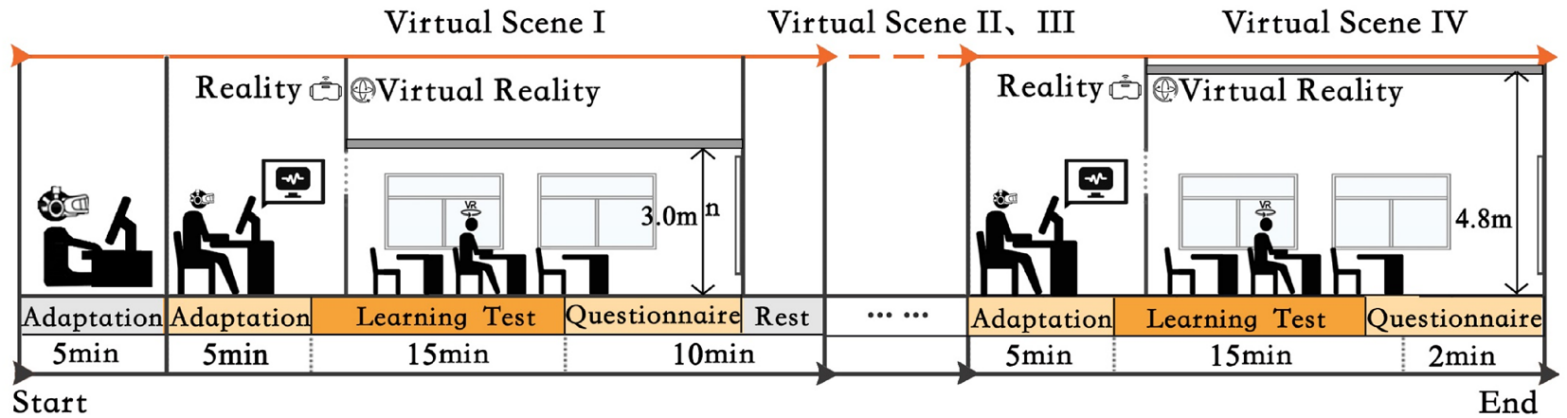
Experiment process by scene.

The experiment lasted 117 min, including a 32-min break, to ensure a balance between participants’ adaptation and fatigue. Participants were able to fully engage in the scenarios without experiencing the exhaustion associated with long periods of testing. Only one participant, as well as an experimental operator, was present during the experiment, and one participant’s experiment was completed in its entirety before the next participant entered the laboratory at the operator’s request. Figure 2 depicts the comprehensive experimental procedure. Prior to commencing the experiment, participants underwent a 5-min period of rest to acclimatise to the equipment. During this time, the experimenter provided an explanation of the procedure and safety measures to the participants. The experiment was subsequently replicated in four randomised virtual scenarios, following the experimental protocol. Participants initially donned VR devices, as well as EEG and electrocardiogram (ECG) devices, to familiarise themselves with the virtual environments. The participants then completed a task test consisting of three types of tests: the Stroop test, calculation test, and reading test (Table 3). Following the initial scene, participants completed the required questionnaire (Table 4). Subsequently, they took a break to prepare for the subsequent scene of the experiment.

**Table 3:** Task test content.

Task	Psychological level	Index	Experiment example
Stroop effect experiment	Perceptual function	Accuracy	Please make sure the accuracy and say the color of the word quickly:
Digital calculation experiment	Thought function	Accuracy	Please make sure the accuracy and calculate the following equation quickly: 337 + 315 = ; 733 − 168 = ; 4 × 237 = ; 12 × 15 =
Reading experiment	Learning and memory functions	Accuracy	Please read and memorize the following text and repeat it in Chinese when you are finished: “Chinese medicine is a heritage of world medicine. Chinese medicine has a power to heal people…”

**Table 4:** Questionnaire seven-level scale.

Type	Level						
	− 3	− 2	− 1	0	1	2	3
Pleasure vote	Very unpleasant	Unpleasant	Slightly unpleasant	Normal	Slightly pleasant	Pleasant	Very pleasant
Relaxation vote	Very nervous	Nervous	Slightly nervous	Normal	Slightly relax	Relax	Very relax
Focus vote	Very unfocused	Unfocused	Slightly unfocused	Normal	Slightly focused	Focused	Very focused
Fatigue vote	Very gentle	Gentle	Slightly gentle	Normal	Slightly fatigue	Fatigue	Very fatigue

### Data analysis

The experimental data consisted of subjective evaluation data, task test data, and physiological parameter data. Prior to conducting data analysis, the data underwent categorization, organization, and the removal of any evident abnormal data, in preparation for subsequent processing.

#### T-score

This paper examines the learning efficiency of participants through three tests: the Stroop test, the Number Crunching test, and the Reading test. All three tests were conducted within a five-minute time frame. Due to the lack of complete uniformity in the three scoring criteria for the tests, there was a significant disparity in the mean scores. To address this, the scores were standardized to ensure comparability. The standardized Z-scores were calculated using Eq. (1), where X represents the raw score for each task test, E(X) represents the expected value, and σ(X) represents the standard deviation. Z-scores represent the deviation from the mean in terms of standard deviation. They quantify the difference between the mean and any individual data point in terms of standard deviation. The Z-score is transformed into a T-score through a linear transformation, as described in Eq. (2), to aid in the interpretation of experimental findings.

1 $${\text{Z}} = \frac{{{\text{X}} - {\text{E(X)}}}}{{\sigma {\text{(X)}}}}$$

2 $${\text{T}} = 50 + 10\left( {\text{Z}} \right)$$

#### Physiological data processing and analysis

1. *ECG data* The ECG data was analyzed for heart rate variability using Kubios software (version: KUBIOS HRV Standard 3.5.0, website link: https://www.kubios.com/). Two HRV metrics were measured: HRV-nLF (low-frequency signal) and HRV-nHF (high-frequency signal). These metrics are linked to sympathetic and parasympathetic activity, respectively. An increase in the low-frequency signal indicates higher mental arousal, while an increase in the high-frequency signal suggests lower mental arousal. The HRV-nLF/nHF level is positively correlated with attention intensity^53^ and is used as a measure of attention intensity in this paper.
2. *EEG *data EEG data must be properly aligned with the EEGLAB (version: eeglab 2021.0 website link: https://sccn.ucsd.edu/eeglab/index.php) analysis by positioning the electrodes before importing it into EEGLAB. A band-pass filter was employed to eliminate artefacts within the frequency range of 0.1–45 Hz, while a suppression filter was utilised to eliminate common frequency artefacts within the range of 48–52 Hz, based on the rhythmic characteristics of the human brain. The data from each segment were analysed using the ICA method to eliminate artefacts such as blinks, horizontal eye movements, and muscle activity that could be misinterpreted as components. The EEG signals of the 34 participants were individually filtered to obtain a valid data set for the experiment. The preprocessed EEG data was analysed using EEGLAB to obtain EEG thermograms and power levels of the EEG beta frequency in different scenarios after participants received environmental stimuli.

In addition, the power values of theta and alpha frequencies can be obtained by the above method and the EEG fatigue level can be calculated by Eq. (3). Where RT_Frontal_ is the theta power value of the frontal lobe of the brain and RA_Parietal_ is the alpha power value of the parietal lobe of the brain.

3 $${\text{EEG fatigue level}} = \frac{{RT_{Frontal} }}{{RA_{Parietal} }}$$

## Results

### Subjective data analysis

**Figure 3:**
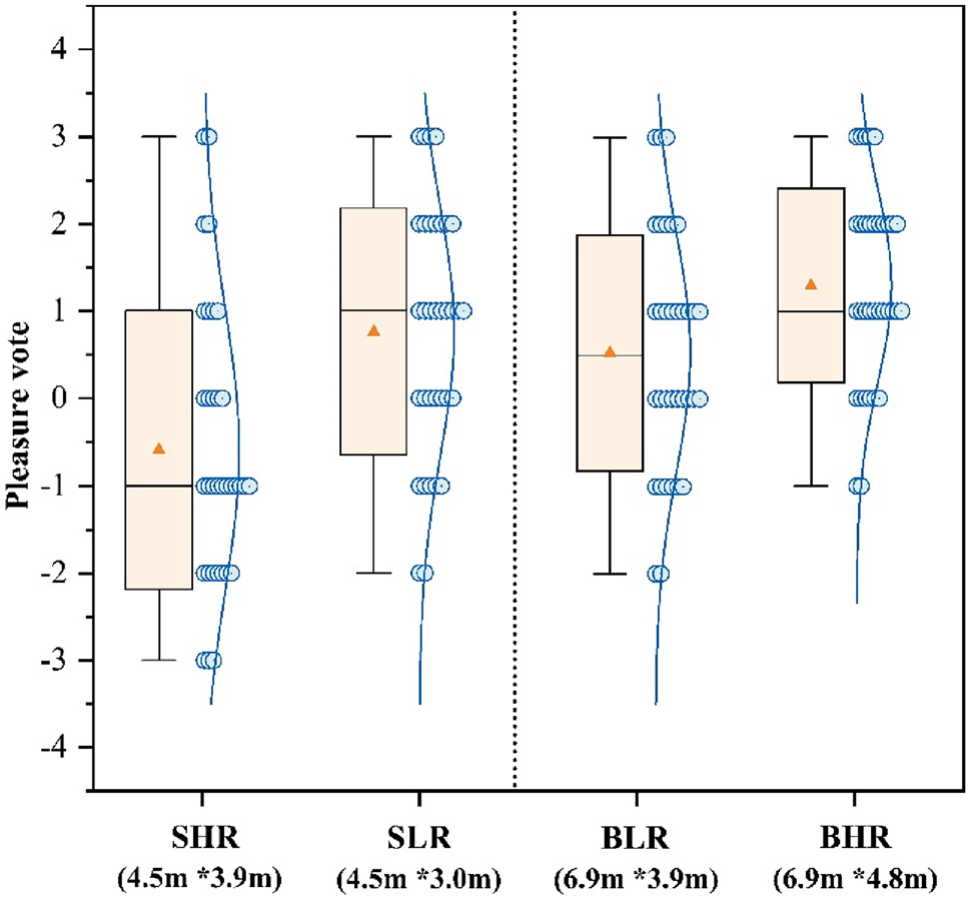
Pleasure vote.

Figure 3 shows the average voting results of people’s ratings of pleasure in the four classrooms. Participants’ subjective evaluation of pleasure varied considerably across classrooms with various sizes and ceiling heights. The BHR classroom had the maximum level of pleasure with a rating of 1.37, whilst the SHR classroom were given the lowest rating of − 0.59. The participants’ subjective rating of pleasure was significantly higher in BLR classrooms as compared to SHR classrooms (*p* < 0.01), indicating that bigger classrooms with identical ceiling height were linked to greater pleasure. The SLR vote in the small classroom was markedly more than the SHR vote. Furthermore, Regarding the SLR, 26.5% of the participants reported feeling “slightly pleasant (+ 1)”. In contrast, in the SHR, only 11.8% felt “slightly pleasant (+ 1)” and 20.6% felt “unpleasant (− 2)”. The BHR vote in big classrooms demonstrated a 0.76 increase compared to the BLR. In the BHR, 14.7% of individuals were classified as “very pleasant (+ 3)”, but in the BLR, this percentage was 8.8%. The findings suggest that smaller classrooms are more pleasant with shorter ceilings, while larger classrooms are more pleasant with higher ceilings.

**Figure 4:**
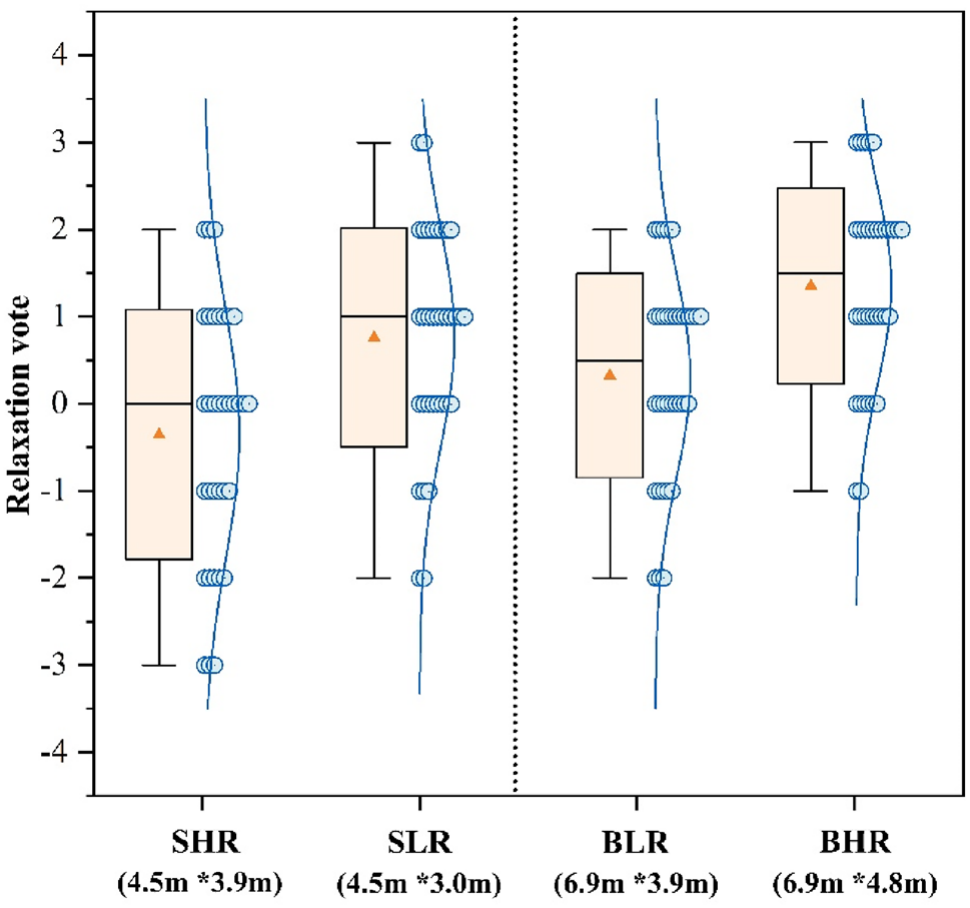
Relaxation vote.

Figure 4 presents the mean voting outcomes for participants’ relaxation levels across the four classrooms. The BHR `classroom had the highest relaxation voting result of 1.35, while the SHR classroom had the lowest relaxation voting result of − 0.35. Participants’ level of relaxation was significantly higher in the BLR classroom compared to the SHR classroom by 0.67 (*p* < 0.01), suggesting that participants experienced greater relaxation in the larger classroom when the ceiling heights were equal. When classrooms were the same size, changes in ceiling height had a significant effect on relaxation voting, where SLR was 1.11 higher than SHR. In SLR, among the participants, 32.4% reported feeling “slightly relax (+ 1)”, compared to 20.6% in SHR. Additionally, 14.7% of participants reported feeling “slightly nervous (− 1)” in SHR. The rating of relaxation for the BHR was voted 1.03 higher than the BLR. In addition, there was a 35.3% greater percentage of those in the BHR who reported feeling “relax (+ 2)” or “very relax (+ 3)” compared to those in the BLR. The findings indicated that individuals had equivalent levels of relaxation in classrooms with smaller sizes and short height, compared to classrooms with bigger sizes and higher ceilings.

**Figure 5:**
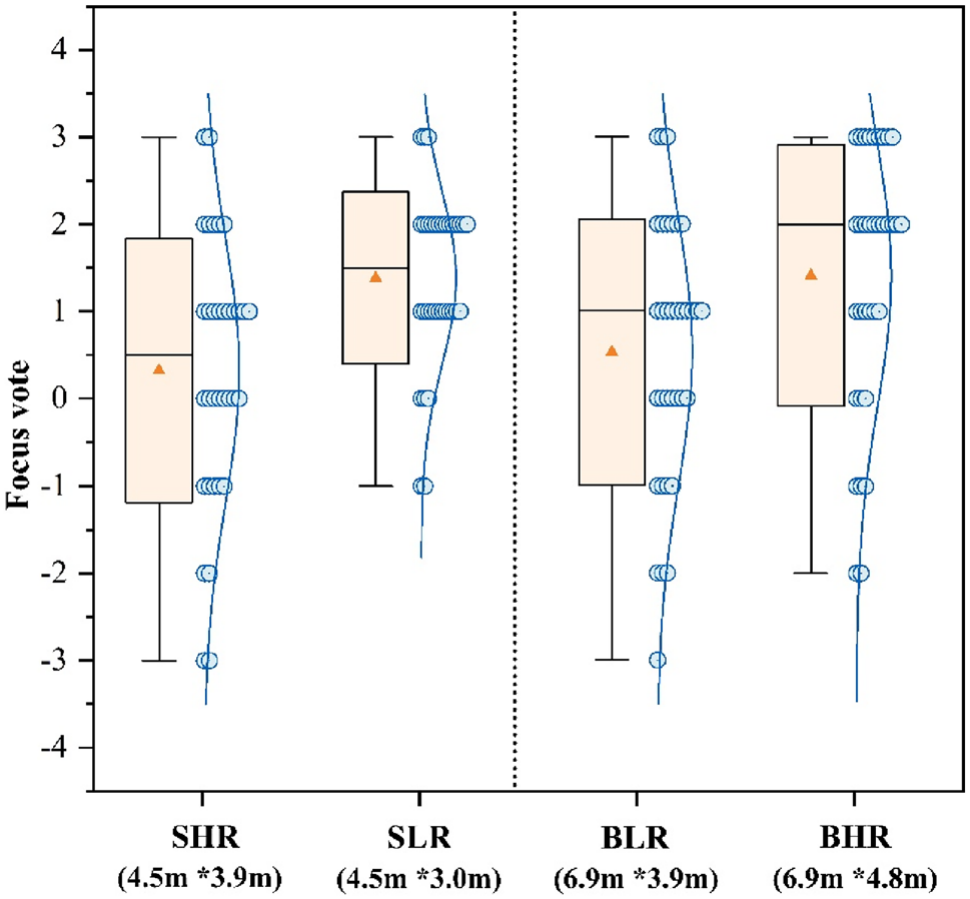
Focus vote.

Figure 5 shows the mean voting outcomes for participants’ focus across the four classrooms. The focus levels of participants in the BHR and SLR classrooms were 1.41 and 1.38, respectively. Although the focus levels were more similar between the two classrooms, 26.5% of participants in the BHR classroom reported feeling “very focused (+ 3)”, compared to only 8.8% of participants in the SLR classroom. This suggests that there is a higher likelihood of participants achieving high focus levels in larger classrooms. The study showed that there was no statistically significant difference in the participants’ focused votes between BLR and SHR (p = 0.71). In the small classroom, the SLR vote was higher than the SHR at 1.06. In the SLR, 35.3% of participants reported “slightly focused (+ 1)”, while in the SHR, this number was 29.4%. The BHR for the big classroom was 0.88 higher than the BLR, and the number of participants rated as “very focused (+ 3)” in the BHR was 17.7% higher than in the BLR. The study found that participants in large classrooms with high ceilings and small classrooms with low ceilings were more focused, with ceiling height having a greater effect than classroom size.

**Figure 6:**
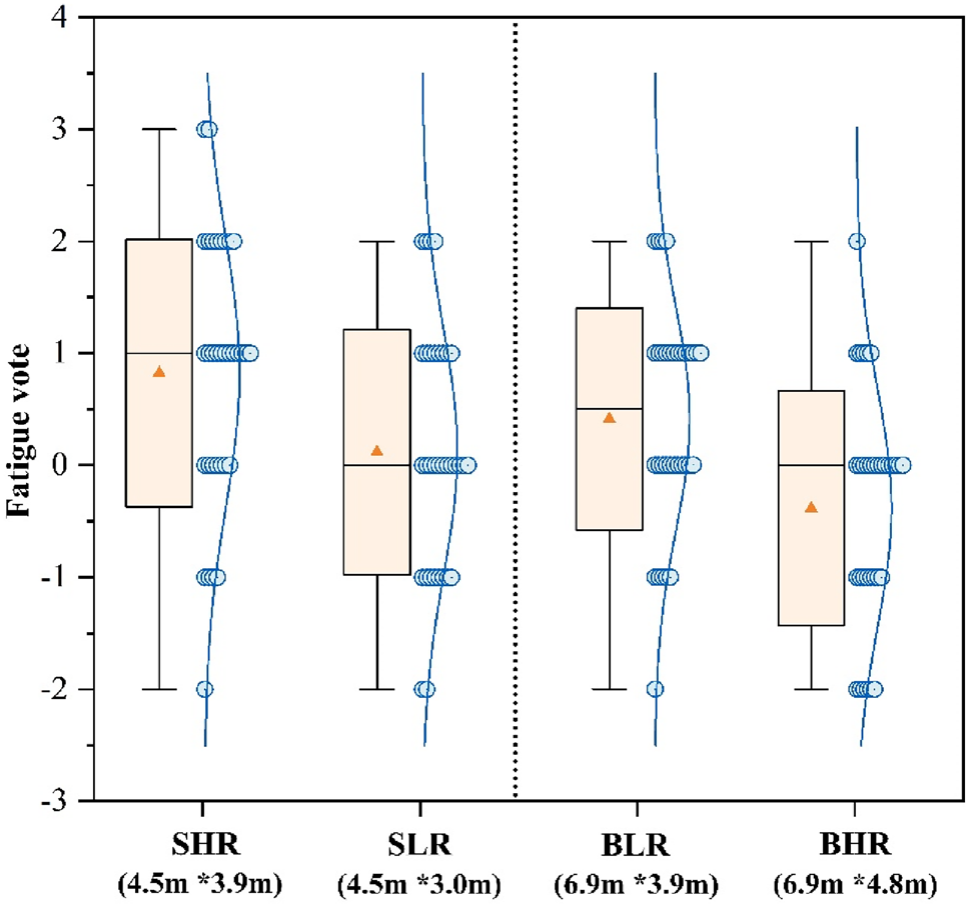
Fatigue vote.

Figure 6 displays the average votes results of participants’ fatigue levels in four different classrooms. The fatigue ratings of participants varied significantly among classrooms with different room sizes and ceiling heights. SHR had the greatest fatigue rating of 0.82, while BHR had the lowest at − 0.38. Participants in the SHR classroom reported feeling significantly more fatigued than those in the BLR classroom by 0.41 (*p* < 0.01). Participants reported more fatigue in the small classroom despite the same ceiling heights. The fatigue rating of SLR in the small classroom was 0.70 lower than that of SHR. 58.8% of SHR participants reported significant “fatigue (+ 2)” or “slightly fatigue (+ 1)” compared to 35.3% in the SLR. Within the context of big classrooms, the BHR vote emerged to be 0.79 less than the BLR. In addition, only 14.7% of the BHR indicated feeling “slight fatigue (+ 1)”, while 23.5% reported feeling less fatigued in comparison to the BLR. The results showed that big classrooms with high ceilings were effective in reducing fatigue in the participants.

## 2Task test score data analysis

**Figure 7:**
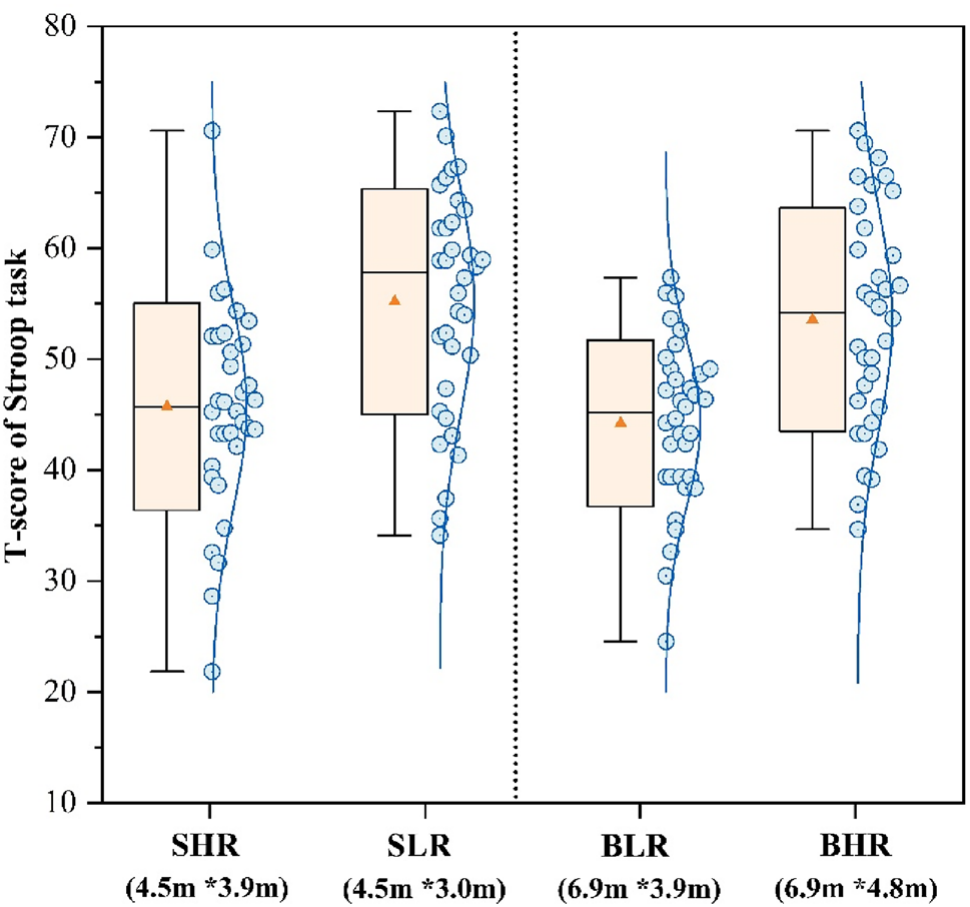
T-score of Stroop task.

**Figure 8:**
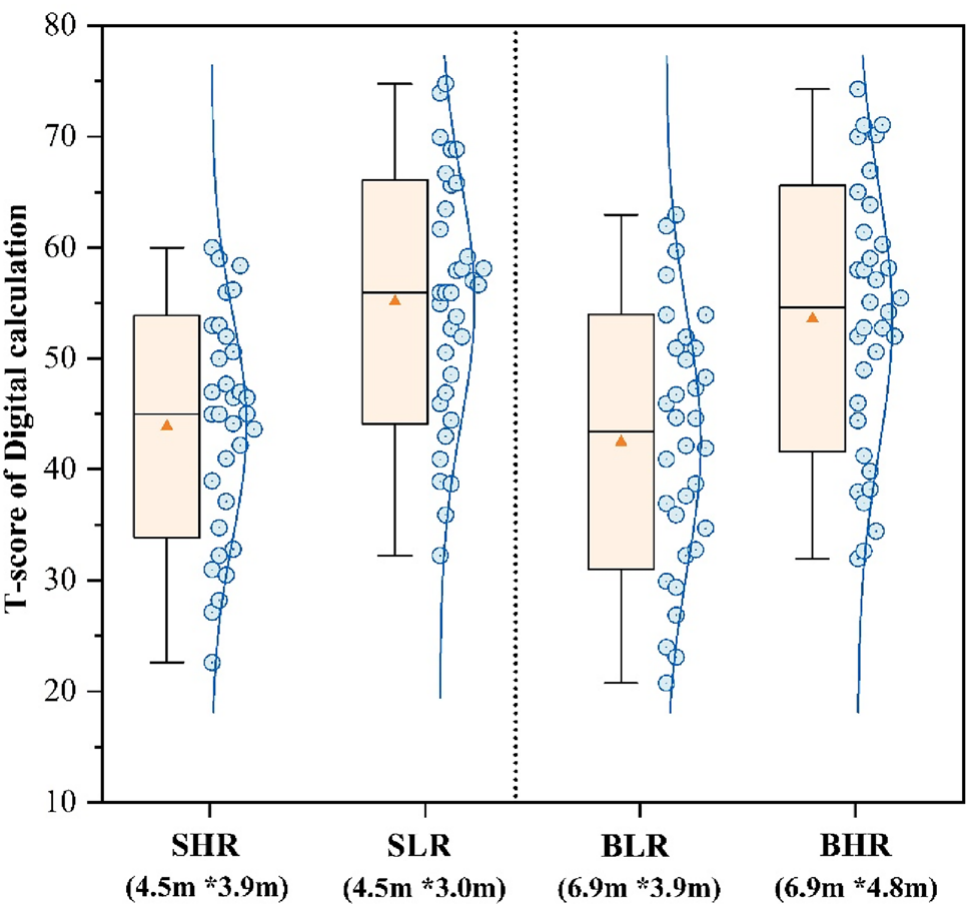
T-score of Digital calculation.

**Figure 9:**
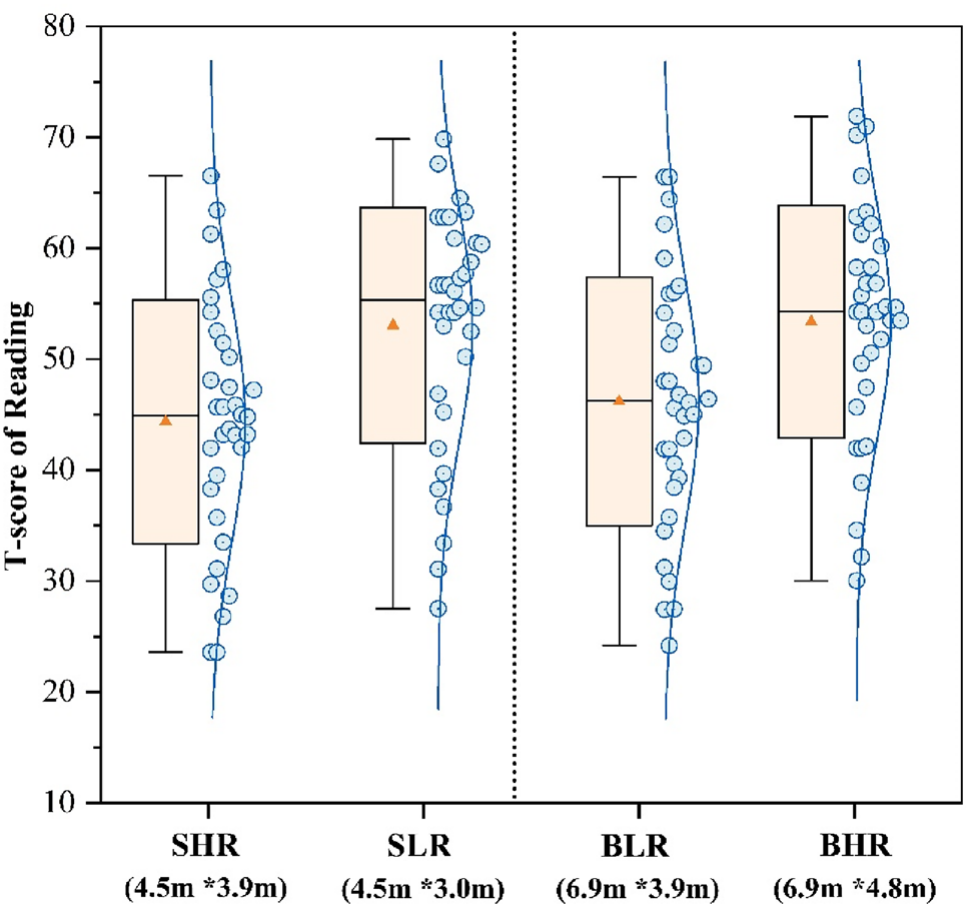
T-score of Reading.

Figures 7, 8 and 9 present the T-scores of the participants on the three task tests across the four scenarios. The SLR classroom had the highest scores in the Stroop test (55.18) and the Digital calculation test (54.42), which were 24.8% and 27.5% higher than the BLR classroom, respectively. The BHR classroom had the highest score in the Reading test (53.81), which was 18.1% higher than the SHR classroom. The alteration in classroom environment had a notable impact on logical thinking abilities, as demonstrated by the Digital calculation test. There was no significant difference in the test scores of participants in both SHR and BLR, indicating that changes in size do not impact test performance. A comparison between small and large classrooms revealed that participants in the small classroom achieved higher scores when the ceiling height was shorter, while the opposite was observed in the large classroom.

**Figure 10:**
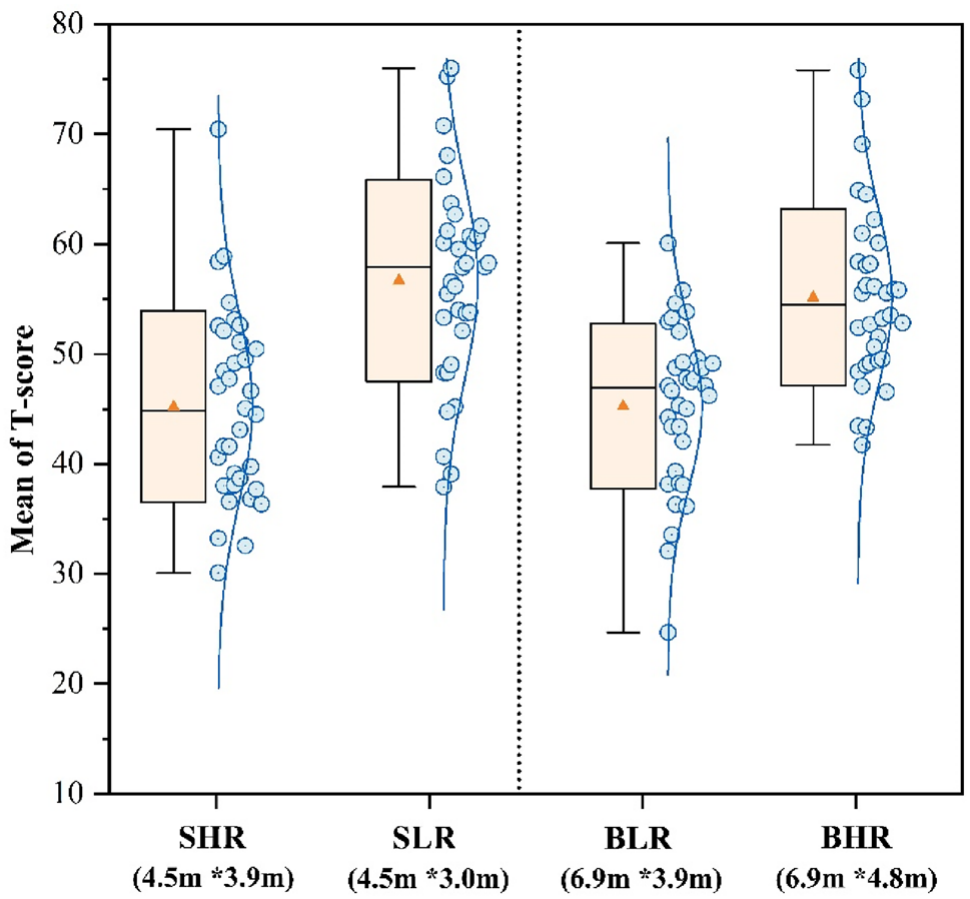
Mean of T-score.

Figure 10 shows the average of the three task scores. The participants in the SLR classroom attained the highest test score of 54.15. In the small classroom, the participants in SLR got scores were 20.1% higher than the SHR scores. In the big classroom, the participants in BHR achieved a score of 52.92, which was 17.3% higher than the scores of the participants in BLR. There was no statistically significant difference in the scores of participants across SLR and BHR classrooms (*p* = 0.61). Therefore, it may be deduced that participants attained higher scores in classrooms of bigger size, with Higher ceilings. Participants in smaller classes with low ceiling had a higher score on tests, especially in cognitive responsiveness and logical thinking.

### Physiological data analysis

The patterns of physiological effects of classrooms at different spatial scales on participants were explored by ECG and EEG, and Pearson correlation analyses were conducted between these two physiological indicators and task test scores to verify the feasibility that ECG and EEG can be used as physiological indicators to demonstrate students’ learning performance.

#### HRV data analysis

**Figure 11:**
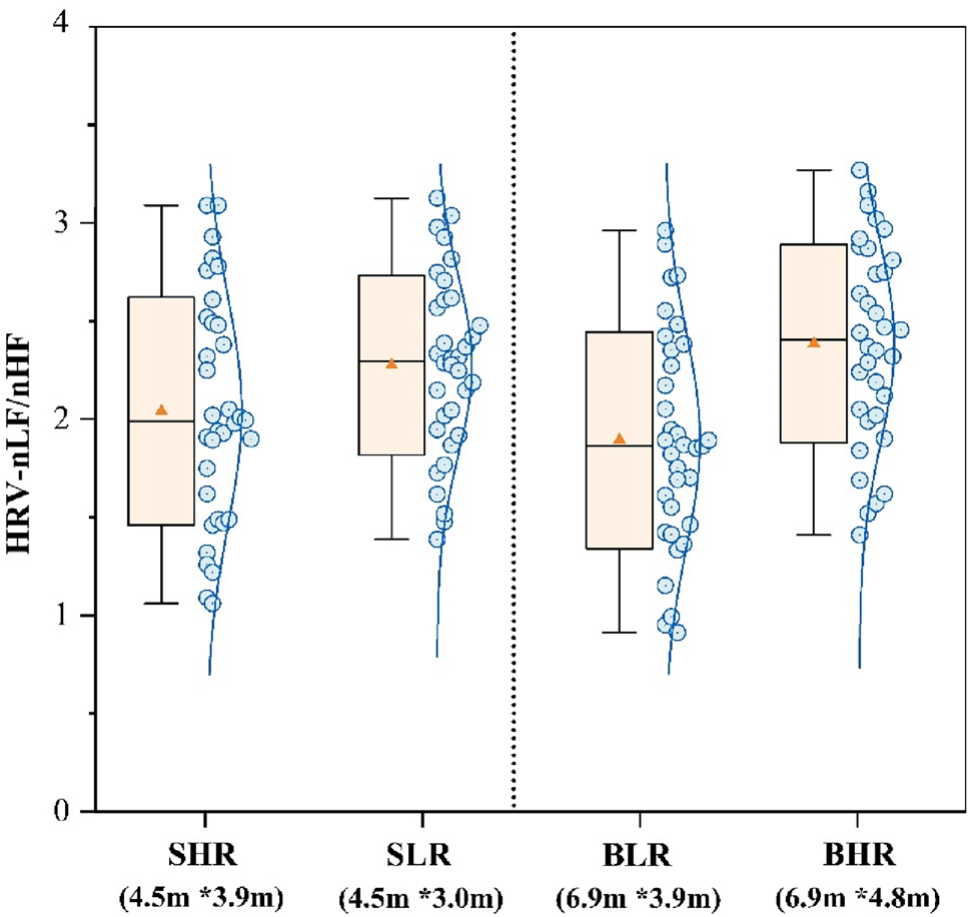
HRV-nLF/nHF of different sence.

Figure 11 shows the HRV- nLF/nHF metrics of the participants’ ECGs under the four scenarios. BLR classroom had the lowest HRV- nLF/nHF of 1.89 Hz, and SLR and BHR classrooms had the highest of 2.28 Hz and 2.39 Hz, which were elevated by 20.6% and 26.5% compared to the BLR classroom, respectively. This physiologically demonstrates that classroom scale changes have an effect on participants’ attentional intensity.

Table 5 shows the Pearson correlation analysis of HRV- nLF/nHF with the raw scores of the task tests, as shown in the table, there is a significant positive correlation between HRV-nLF/nHF and the composite scores of the three task tests, thus indicating that electrocardiographic HRV-LF/HF can be used as a physiological indicator to demonstrate the learning performance of students (Table 6).

**Table 5:** Correlation analysis between task test performance and HRV-LF/HF in the 34 participants.

		Stroop effect experiment	Digital calculation experiment	Reading experiment
HRV-LF/HF	Pearson correlation	0.62**	0.79*	0.83**
	Sig. (2-tailed)	< 0.01	< 0.05	< 0.01

*p<0.05, **p<0.01

**Table 6:** Active point (Ep) frequency statistics for the 34 participants.

Region									
Frontal		Occipital		Parietal		Left temporal		Right temporal	
Channel	Count	Channel	Count	Channel	Count	Channel	Count	Channel	Count
Fp2	63	CP2	35	P4	19	FC5	16	TP10	11
Fp1	57	FC2	34	Pz	15	FT9	14	CP6	9
Fz	26	FC1	22	P3	15	T7	11	FT10	5
F4	23	C4	19	P7	15	TP9	8	T8	1
F7	21	Cz	12	P8	14	CP5	5	FC6	–
F3	14	CP1	1	O1	9				
F8	10	C3	1	O2	5				
				Oz	–				

#### EEG data analysis

**Figure 12:**
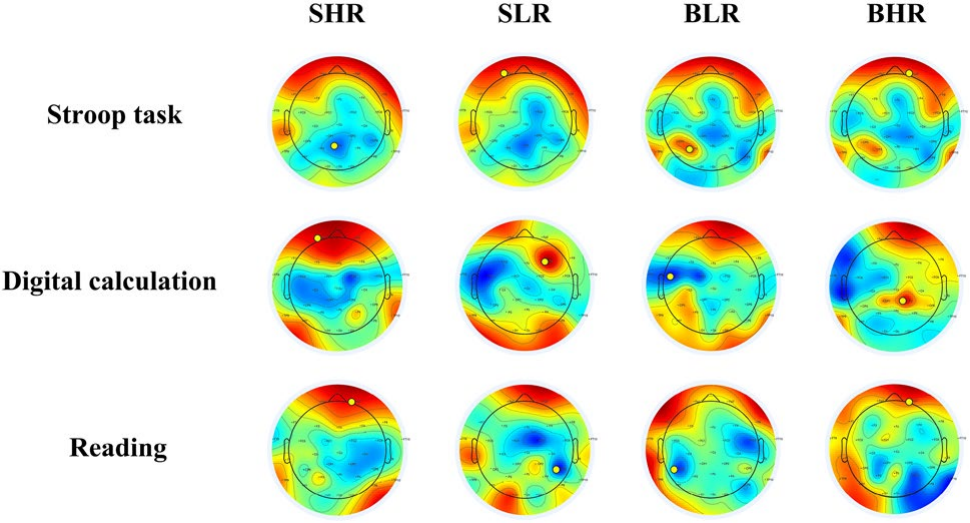
Topographic map of the EEG -β frequency of one participant.

EEG is capable of characterising alterations in electrical activity observed on the surface of the scalp^54^. EEG can be classified based on frequency into delta (δ, 1–4 Hz), theta (θ, 5–7 Hz), alpha (α, 8–13 Hz), beta (β, 14–30 Hz), and gamma (γ, 30 Hz and above)^55^. Previous research has found a strong positive relationship between the beta frequency of EEG and attention intensity^56,57^. This chapter focuses on the regions of the brain and channel locations that have the highest levels of β frequency in conditions of experimentation. The study does not track the EEG fatigue index calculations, which indicate the specific regions and channels that are under investigation. The purpose of this study is to evaluate the impact of different classrooms on levels of participants’ attention and fatigue. The study collected brainwave thermograms from 34 participants in various scenarios and task tests. The channel with the highest energy value in each thermogram was recorded as the Ep point. In total, there were 510 Ep points (Table 6). Figure 12 displays the energy magnitude of three task tests conducted by participant 15 in four different environments. Darker shades of blue or red in the heat map represent greater power (the positive and negative power only represents the positive and negative voltage, not the magnitude of the power)^58,59^. The figure displays the Ep points for participant 15 in the Stroop test, indicating Pz, FP1, P3, and FP2 as the locations. The Ep points assigned to the four scenarios in the Digital calculation test are FP1, F4, FC5, and CP2. The Ep points assigned in the Reading test are FP2, CP6, FC5, and FP2. The channels Fp1, Fp2, CP2, and FC2 exhibit a high level of active β-frequencies and are situated in the frontal and occipital lobes. Thus, the brain activity of the participants was compared using four channels.

**Figure 13:**
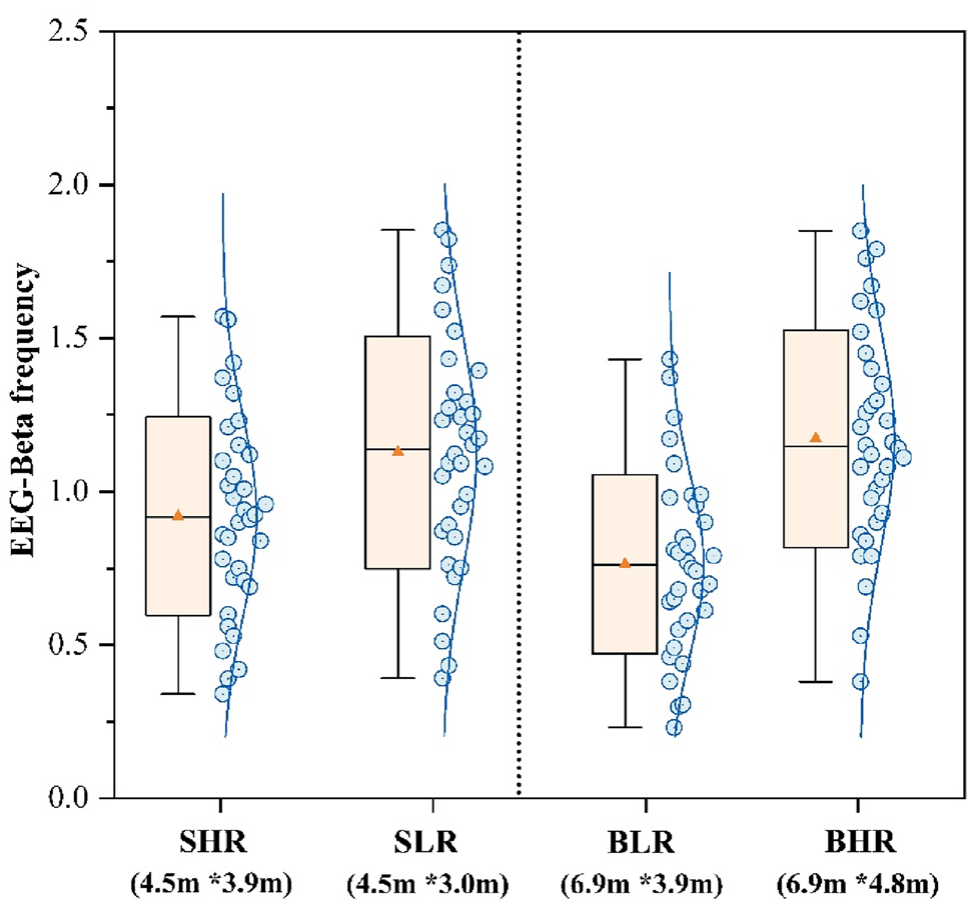
EEG-β of different sence.

Raw electrical signals collected by EEG equipment are preprocessed and Fourier transformed to obtain power values (μv) in the β-frequency domain, and this method is commonly used to demonstrate a participant’s attentional intensity^60^. In addition, a large number of studies have demonstrated a significant correlation between EEG-β and attentional intensity^61^. The study investigated the mean beta frequency power levels of the participants’ EEG Fp1, Fp2, CP2, and FC2 channels in four different scenarios, as shown in Fig. 13. The mean β-frequency power of participants in the SLR classroom was 1.13 μv, indicating a 22.8% rise compared to SHR. The mean beta frequency power of individuals in the BHR classroom was 1.17 μv, indicating a 53.9% rise in comparison to BLR. Participants demonstrated higher beta frequency power when exposed to big classrooms with high ceilings, as well as small classrooms with low ceilings.

**Figure 14:**
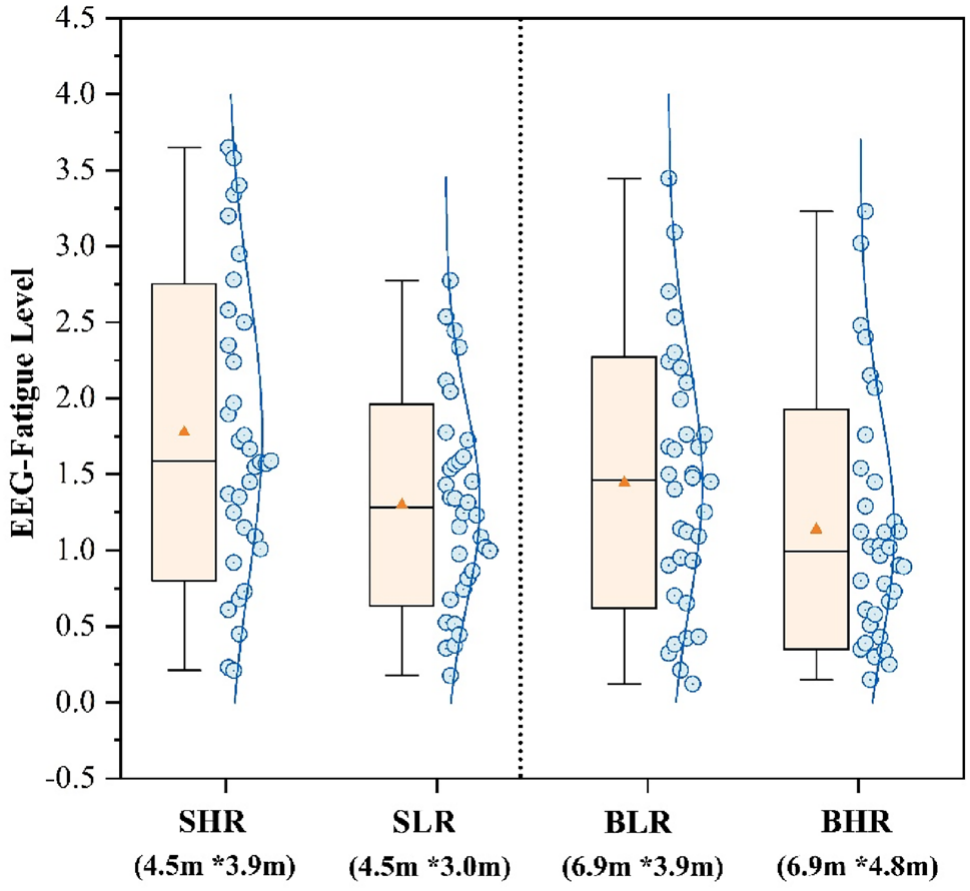
EEG fatigue level of different sence.

Figure 14 displays the EEG fatigue levels of the participants in four scenarios. The theta average power values of specific channels in the frontal lobe (Fp2, Fp1, Fz, F4, F7, F3, F8) and the alpha average power values of specific channels in the parietal lobe (CP2, C4, FC1, FC2, Cz, CP1, C3) were chosen based on the formulae study. The figure shows that the participants in SHR classroom had the highest EEG fatigue index at 1.78, while the participants in BHR classroom had the lowest result at 1.14. The EEG fatigue index of participants in the SLR classroom decreased by 27.0% compared to the SHR, while the EEG fatigue index of participants in the BLR classroom increased by 26.3% compared to the BHR. The EEG fatigue index of participants in SHR experienced a 23.6% increase in comparison to the BLR. This study illustrates that participants experience the lowest levels of fatigue within big classrooms with high ceilings and small classrooms with low ceilings. The result is consistent with the subjective evaluation of weariness.

Table 7 shows the Pearson correlation analysis between EEG-β and raw scores of the task tests, as shown in the table, there is a significant positive correlation between EEG-β and the composite scores of the three task tests, thus suggesting that EEG-β can be used as a physiological indicator to prove the learning performance of students.

**Table 7:** Correlation analysis between task test performance and EEG rhythm in the 34 Participants.

		Stroop effect experiment	Digital calculation experiment	Reading experiment
EEG-β	Pearson correlation	0.76**	0.85**	0.69**
	Sig. (2-tailed)	< 0.01	< 0.01	< 0.01

*p<0.05, **p<0.01

## Discussion

The objective of this study is to confront the methodological challenges which are technically in nature and have limited the amount of research conducted on indoor environment design, specifically evidence-based design (EBM, evidence-based medicine) in classrooms. As paired with techniques based on the gathering of EEG and ECG measurements, immersive VR technology makes it possible to precisely control a specific architectural variable. This allows for the collection of objective neurological data from participants while they complete task assessments in different environments. In the experiment, four different classrooms were designed using VR technology, and a total of 34 college students were given the task test, during which physiological parameters were recorded and questionnaires were completed to explore the effect of room size and ceiling height on the students’ learning performance, and to provide theoretical support for the design of future learning spaces.

Figure 3 shows that big classrooms with high ceilings have the highest results in the pleasure vote, this result is in accordance with the findings of many past studies. For example, Stamp et al.^13^ found through a questionnaire that people tend to experience more positive emotions in more open environments. Franz et al.^62^ found that the degree of openness of a room affects people’s experience of architectural space, especially in terms of emotional responses such as “interesting” and “happiness”. Figure 4 shows that participants were more relaxed in a big classroom with a high ceiling, combined with Fig. 6, it shows that fatigue was the lowest in this environment. Thus in comparison to other classrooms, participants in big classrooms with high ceilings were more likely to show positive emotional responses and thus recover from fatigue, and a similar trend has been observed in related architectural studies^63^.Meanwhile participants in big classrooms with low ceilings had the highest fatigue voting results (Fig. 6), but small classrooms with low ceilings did not have a negative effect on the participants, but instead the participants rated small classrooms with low ceilings higher compared to small classrooms with high ceilings. For this reason some scholars have done studies, for example, Ching, F.D et al.’s study^64^ concluded that low height ceilings have a greater negative impact on large rooms compared to small rooms. This is mainly due to the fact that low ceilings create a feeling of oppression, while people feel comfortable facing the pressure from the ceiling instead when they are in small-sized rooms^19^.

As the results of the Fig. 10 task test showed that changes in ceiling height had a significant effect on participants’ performance, which is similar to the results of studies conducted by other scholars^11,65^, it is worthwhile for designers to consider that ceiling height is an important variable that affects students’ performance. Students performed best in small classrooms with low ceilings, but it is remarkable that many students performed just as well, or even better, in big classrooms with high ceilings than in other environments. In contrast, research by Moore, G.T., et al.^24^, by comparing student performance in classrooms of different heights, suggests that students’ learning behaviours are more restrained and focused in classrooms with low ceiling heights, while classrooms with high ceilings may lead to more socially active behaviours. In this study, the high ceiling focus vote (Fig. 5) was higher in the big classroom, and the reason for feeling more focused may be related to the comfort level, which has been suggested by some studies^66,67^ to make it easier for people to focus on their studies in a comfortable space than in an uncomfortable space. In a follow-up investigation, it was found that students who performed best in big classrooms with high ceilings had significantly higher GPAs than other students, and their performance on the task tests in the experiments tended to be stable and susceptible to the “ceiling effect”, i.e., they were less affected by changes in the environment. In addition, the results of the study showed that the change in the size of the classroom had a smaller effect on the participants’ performance on the task test than the change in height, with no statistical difference, which may be related to the shorter testing time of the participants, and the type of tasks can be improved in the subsequent experiments. Apparently, small and low classrooms are more conducive to improving students’ performance than big and tall classrooms. As in the cathedral effect, big and tall classrooms are more suitable for Learning activities that require imagination, for example, reading, a Learning activity that requires thinking logically, is more suitable to be conducted in a small and low classroom.

Figure 11 shows that changes in ceiling height had a significant effect on participants’ HRV indicators, whereas it has been previously demonstrated that changes in HRV are closely related to participants’ neurological activity, and that an increase in HRV- nLF/nHF represents an increase in the intensity of focus^37^. And the present study found a significant positive correlation by correlating HRV- nLF/nHF with the scores of the three task tests (Table 5), which further demonstrated that HRV- nLF/nHF can be used as a physiological indicator to quantify participants’ learning performance. However, notably, the difference in the results of HRV- nLF/nHF was not as significant as the difference in the results of Fig. 5, which might be related to the fact that the duration of the task test per person was only 15 min, resulting in a short physiological acquisition time. The EEG thermograms from participants completing three task tests in four classrooms were examined in this work to identify the channels with the most significant changes in order to target and analyse the pattern of change in EEG-β power. The frontal and occipital lobes’ channels Fp1, Fp2, CP2, and FC2 were closely associated with neurological activity when the participants completed the task tests, as Table 6 showed. However, due to the limited number of samples, this study was unable to identify any patterns between the various task test types and the presence of an active distribution of EEG-β power values.

It is worth noting that it is not only the participants’ perception of space that affects their mood, but also the degree to which they are successful in their tasks test. And this influencing process itself comes back to the participants’ learning performance. Therefore, to investigate the correlation between participants’ subjective evaluations and learning performance, Pearson correlation analyses were conducted on the participants’ subjective evaluations and the three task tests. As shown in Table 8, there is a significant positive correlation between the voting results of the subjective evaluation of pleasure and relaxation and the scores of the three task tests, which indicates that when the participants are more efficient in learning, they have a higher subjective evaluation of the comfort of the scene, especially the score of the digital calculation test and the voting results of the pleasure vote show a strong correlation (R^2^ = 0.87). Subjective evaluation of concentration was positively correlated with each of the three tests, especially with the Stroop effect test score and the subjective concentration vote (R^2^ = 0.93), indicating that participants’ cognitive responses are highly susceptible to concentration. The Pearson’s coefficients of the subjective evaluation of fatigue and the three tests are R^2^ =  − 0.48, R^2^ =  − 0.55, R^2^ =  − 0.47, which suggests that when subjects feel fatigued with the scene, they may have a lower learning performance.

**Table 8:** Correlation analysis between task test performance and EEG rhythm in the 34 Participants.

		Stroop effect experiment	Digital calculation experiment	Reading experiment
Pleasure vote	Pearson correlation	0.78**	0.87**	0.63**
	Sig. (2-tailed)	< 0.01	< 0.01	< 0.01
Relaxation vote	Pearson correlation	0.63**	0.74**	0.75**
	Sig. (2-tailed)	< 0.01	< 0.01	< 0.01
Focus vote	Pearson correlation	0.93**	0.89**	0.89**
	Sig. (2-tailed)	< 0.01	< 0.01	< 0.01
Fatigue vote	Pearson correlation	− 0.48**	− 0.55**	-0.47**
	Sig. (2-tailed)	< 0.01	< 0.01	< 0.01

*p<0.05, **p<0.01

### Limitations

This study’s sample population had thorough screening to prevent participants from being uncomfortable in the virtual environment or unfamiliar with the VR equipment, which could have affected the experimental results. As a result, the experimental sample was somewhat limited, which could have raised the risk of a type II error (false-negative findings).

In order to collect participants’ subjective evaluations of the classroom after the completion of the learning activities, the pleasure and relaxation questionnaires were given out at the end of the task test. In future studies, pleasure and relaxation questionnaires can be added once before the task test begins, and by comparing the questionnaires before and after the task test, it can be investigated whether there are differences in participants’ subjective evaluations before and after the task test in different environments, and what the patterns of the differences are.

In this study, the “ceiling effect”, where many participants scored near-perfect on the task, limits some of the conclusions that can be drawn about the effects of these different environments. Ultimately, there are a number of related variables, such as the deployment of personnel in the virtual environment and whether there should be a teacher or other students in the classroom, that were not assessed in the study.

In addition, the study was conducted in a virtual environment. Although numerous studies have demonstrated that differences in behavioral and cognitive responses between participants in VR environments and real environments are minimal, some differences between virtual environments and real environments are inevitable. Future research could enhance the rigor and credibility of the experiment by including comparative studies between virtual and real environments.

## Conclusion

This study examines the subjective evaluation and objective physiological measures of college students during task tests. It analyses the impact of classroom sizes and ceiling heights on learning performance. The following findings were derived:

- (1) The subjective ratings were significantly higher in the large classroom compared to the small classroom. The BHR classroom exhibited significantly higher levels of pleasure, relaxation, and concentration (1.37, 1.35, 1.41) compared to the SHR classroom, which had notably lower levels of pleasure, relaxation, and concentration (− 0.59, − 0.35, 0.32). Additionally, the BHR classroom had the lowest fatigue (− 0.38), while the SHR classroom had the highest fatigue (0.82).
- (2) There was no significant difference in the effect of room size change on task test scores when space heights were the same. Participants in the SLR and BHR groups shown a respective increase of 20.1% and 17.3% in the tests compared to the participants in the other two rooms, which were of the same size. Participants’ test scores in SLR and BHR classrooms were comparable and higher, with no statistically significant difference (*p* > 0.05). Participants’ test scores in SLR had higher scores (54.14) compared to the SHR by 20.1% and participants’ test scores in BHR scores (52.92) by 17.3% over BLR.
- (3) The ECG results indicated that the participants’ HRV-nLF/nHF was higher and less varied during the SLR classroom and the BHR classroom, with values of 2.28 Hz and 2.39 Hz respectively. These values were 20.6% and 26.5% higher compared to the SHR classroom, which had the lowest results. Pearson correlation analysis was conducted to examine the relationship between HRV-nLF/nHF and the three task tests. The results showed a significant positive correlation.
- (4) The EEG results indicated that there was a higher beta frequency power in the SLR and BHR classrooms, but the difference was not statistically significant. The SLR classroom exhibited a 22.8% increase in beta frequency power (1.13 μv) compared to the SHR. Similarly, the BHR classroom showed a 53.9% increase in mean beta frequency power (1.17 μv) compared to the BLR. The BHR classroom exhibited the lowest EEG fatigue metrics (1.14), indicating a 36.0% decrease compared to the SHR classroom, which had the highest results. The Pearson correlation analysis revealed a significant positive correlation between beta frequency power values and the scores of the three task tests.

## Data Availability

Because the overall experiment of this study has not been fully concluded, the data sets generated in this study have not been publicly available, but are available to the corresponding authors upon reasonable request.
